# Supplementary material for: Non-Biopsy Serology-Based Diagnosis of Celiac Disease in Adults Is Accurate with Different Commercial Kits and Pre-Test Probabilities
Source: Nutrients. 2020 Sep 8;12(9):2736. doi: 10.3390/nu12092736 (PMC7551634; doi:10.3390/nu12092736)
Supplement: Supplementary file 1 [file nutrients-12-02736-s001.pdf]

Table S1. Characteristics of the 15 subjects who received a celiac disease diagnosis after special investigations and a trial with gluten-free diet (GFD).

Age, years	Sex	HLA	Symptoms		TGA, U/ml[2]		EmA, titer[4]		VH/CrD		CD3+ IELs[5]		γδ+ IELs[1]	IgA dep.
			At dg	GFD	At dg	GFD	At dg	GFD	At dg	GFD	At dg	GFD		
**Clinical cohort**														
60	F	DQ2	Diarrhea, bloating	None	48.2	1.9	1:1000	Neg	2.8	3.0	84	44	13.8	Yes
58	F	DQ2	Bloating	Improved	10.3	1.4	1:50	Neg	2.4	2.9	55	36	25.9	Yes
42	M	DQ2/8	Abdominal symptoms	None	4.2	3.7	1:100	1:5	2.4	3.4	65	24	14.0	Yes
57	F	DQ2	Abdominal pain	Improved	9.0	0.6	1:100	Neg	2.4	3.8	56	44	39.2	Yes
22	F	DQ2	Abdominal symptoms	Improved	2.7	0.6	1:5	Neg	2.4	3.2	88	52	38.5	Yes
70	F	DQ2/8	Bloating, anemia	Improved	25.7	2.0	1:500	Neg	2.5	3.5	134	103	31.8	Yes
61	M	DQ2/8	Diarrhea, weight loss	Improved	1.7	0.0	1:5	Neg	3.3	3.7	37	33	8.3	Yes
57	F	DQ2/8	Bloating	None	4.9	1.6	1:5	Neg	2.8	3.3	39	38	7.0	Yes
45	F	DQ2	Abdominal symptoms	Improved	4.7	0.6	1:5	Neg	2.3	2.8	73	53	31.8	Yes
39	M	DQ2	Abdominal symptoms	None	5.7	0.3	1:5	Neg	2.2	4.3	62	31	26.5	Yes
44	M	DQ2	Diarrhea, bloating	Improved	6.8	2.1	1:50	Neg	3.0	3.3	59	29	19.1	Yes
26	M	DQ2	Bullous rash*	None	21.6	ND*	1:200	ND*	2.7	ND*	50	ND*	39.0	Yes
**Family cohort**														
80	F	DQ2	Bullous rash*	None	165[3]	ND*	1:50	ND*	ND	ND	ND	ND	ND	ND
34	M	DQ2	Tiredness	None	56[3]	0.0	1:5	1:<5	2.2	2.7	72	52	16.9	Yes
30	F	DQ2	Flatulence, bloating	Improved	84.1	7.6	1:500	1:100	3.4	2.8	79	76	43.9	Yes

[1]Cells/mm, reference value <4.3; [2]Celikey; reference value <5.0 U/ml, except [3]Inova, reference value <20 U/ml; [4]Reference value 1: <5; [5]Cells/mm, reference value <37. EmA, anti-endomysial antibodies; HLA, human leukocyte antigen; IEL, intraepithelial lymphocytes; IgA dep., transglutaminase 2 specific intestinal IgA deposits; ND, not defined: TGA, anti-transglutaminase 2 antibodies; VH/CrD, villus height crypt depth ratio, reference value ≥2.0. *Skin biopsy confirmed dermatitis herpetiformis diagnosis and no repeat biopsy was made.

Table S2. Positive predictive values (PPV) of the four study tests for celiac disease in the clinical and family cohorts when counting only those cases receiving the diagnosis in routine histology ("worst case scenario").

	≥10x ULN[1]				≥1x ULN[1]			
	Positive subjects	Celiac disease	PPV	95% CI	Positive subjects	Celiac disease	PPV	95% CI
	**n**	**n**	**%**	**%**	**n**	**n**	**%**	**%**
**Clinical cohort**								
Celikey	56	56	100	92.0-100	116	110	94.8	88.6-97.9
Orgentec	36	36	100	88.0-100	113	101	89.4	81.8-94.2
Eurospital	51	50	98.0	88.2-99.9	121	101	83.5	75.4-89.4
Inova	54	53	98.1	88.8-99.9	134	103	76.9	68.6-83.5
**Family cohort**								
Celikey	18	18	100	78.1-100	66	64	97.0	88.5-99.5
Orgentec	26	26	100	84.0-100	78	71	91.0	81.8-96.0
Eurospital	33	33	100	87.0-100	84	76	90.5	81.6-95.5
Inova	21	21	100	80.8-100	93	82	88.2	79.4-93.7

[1]Celikey 5.0 U/ml; Inova 20 U/ml; Orgentec 10 U/ml; Eurospital 10 U/ml
CI, confidence interval; ULN, upper limit of normal
